# Alterations of the gut microbiome and metabolic profile in CVB3-induced mice acute viral myocarditis

**DOI:** 10.1186/s12866-023-02863-4

**Published:** 2023-05-18

**Authors:** Qing Kong, Lili Chen, Xiaochun Zeng, Feiyu Lu, Yanlan Huang, Weifeng Wu

**Affiliations:** Department of Cardiology, the First Affiliated Hospital of Guangxi Medical University, Nanning, China

**Keywords:** Gut microbiota, Metabolomics, Omics integration, Metabolic pathways, Viral myocarditis

## Abstract

**Background:**

Acute viral myocarditis (AVMC) is an inflammatory disease of the myocardium. Evidence indicates that dysbiosis of gut microbiome and related metabolites intimately associated with cardiovascular diseases through the gut-heart axis.

**Methods:**

We built mouse models of AVMC, then applied 16 S rDNA gene sequencing and UPLC-MS/MS metabolomics to explore variations of gut microbiome and disturbances of cardiac metabolic profiles.

**Results:**

Compared with Control group, analysis of gut microbiota showed lower diversity in AVMC, decreased relative abundance of genera mainly belonging to the phyla Bacteroidetes, and increased of phyla Proteobacteria. Metabolomics analysis showed disturbances of cardiac metabolomics, including 62 increased and 84 decreased metabolites, and mainly assigned to lipid, amino acid, carbohydrate and nucleotide metabolism. The steroid hormone biosynthesis, cortisol synthesis and secretion pathway were particularly enriched in AVMC. Among them, such as estrone 3-sulfate, desoxycortone positively correlated with disturbed gut microbiome.

**Conclusion:**

In summary, both the structure of the gut microbiome community and the cardiac metabolome were significantly changed in AVMC. Our findings suggest that gut microbiome may participate in the development of AVMC, the mechanism may be related to its role in dysregulated metabolites such as steroid hormone biosynthesis.

**Supplementary Information:**

The online version contains supplementary material available at 10.1186/s12866-023-02863-4.

## Introduction

Acute viral myocarditis (AVMC) is a common cardiovascular disease, characterized by virus-triggered myocardial inflammation, and followed by autoimmunity. Patients with AVMC may recover or progress to dilated cardiomyopathy (DCM), which can suffer from heart failure and sudden death [1]. Enteroviruses especially Coxsackievirus group B type 3 (CVB3) is known to be one of the dominant causes factors for AVMC, and is taken into esophageal and gastrointestinal tracts via fecal-oral route [2]. Nevertheless, although supportive care or immuno-suppressive therapies are undoubtedly effective, lots of AVMC patients still suffer from sudden death or progress to end-stage cardiac disease, DCM [1]. Therefore, the exact pathogenesis of AVMC is urgently needed to explore and improve novel therapeutics on disease intervention.

Patients with myocarditis frequently present with nonspecific systemic symptoms such as diarrhea, nausea, vomiting, fever and sore throat, sort of like manifestations of gastroenteritis. Enterovirus CVB3 can spread into blood and infect myocardiocytes after evading and proliferating in the intestine [3]. As a result, the gut, which is the initial infection place for CVB3 in AVMC, needs to be investigated [4].

The human gut microbiome harbors trillions of microbes [5], and its genome is 150 times larger than the identified human genome [6–8]. Accumulative evidence shows that the host metabolism could be affected by manipulation of the gut microbiota composition. Furthermore, a growing body of evidence uncovers that gut microbiota produce plenty of metabolites, some of which are biologically active in systemic circulation and play an important role in host [9, 10]. For example, gut microbiota-dependent metabolite trimethylamine N-oxide (TMAO) promotes atherosclerosis, participates in the pathologic processes of heart failure (HF) and Cardiovascular disease (CVD) [11, 12]. In addition, it has been reported that microbiota derived short chain fatty acids (SCFA) protects against cardiac hypertrophy, fibrosis, vascular dysfunction, and hypertension [13]. Thus, through metabolism-dependent pathways, the gut microbiome can communicate with distal organs and play important function. Furthermore, supported by accumulating data of omics integration, the gut microbiota and its metabolites were regarded as a novel therapeutic target for kinds of diseases, such as chronic kidney disease [14], colorectal cancer [15], atopic dermatitis [16], atherosclerosis [17], hypertension [18], heart failure [19]. Thus the contribution of microbiome and its metabolome to cardiovascular diseases, and the existence of gut-heart axis were confirmed [20, 21].

Cardiac inflammation of experimental autoimmune myocarditis (EAM) was induced by bacterial peptide mimics, which was derived from the intestinal microbiota [22]. Notably, Xiao-Fan Hu et al. reported that increased of microbial richness, diversity and Firmicutes/Bacteroidetes ratio in the EAM mice [23]. These data demonstrated that EAM was associated with gut microbiota dysbiosis. Except EAM, CVB3-induced AVMC, is one of the widely accepted and most commonly used animal models of myocarditis [24, 25]. However, until now, the interplay between metabolites and gut microbiota has not been explored in AVMC.

**Fig. 1:**
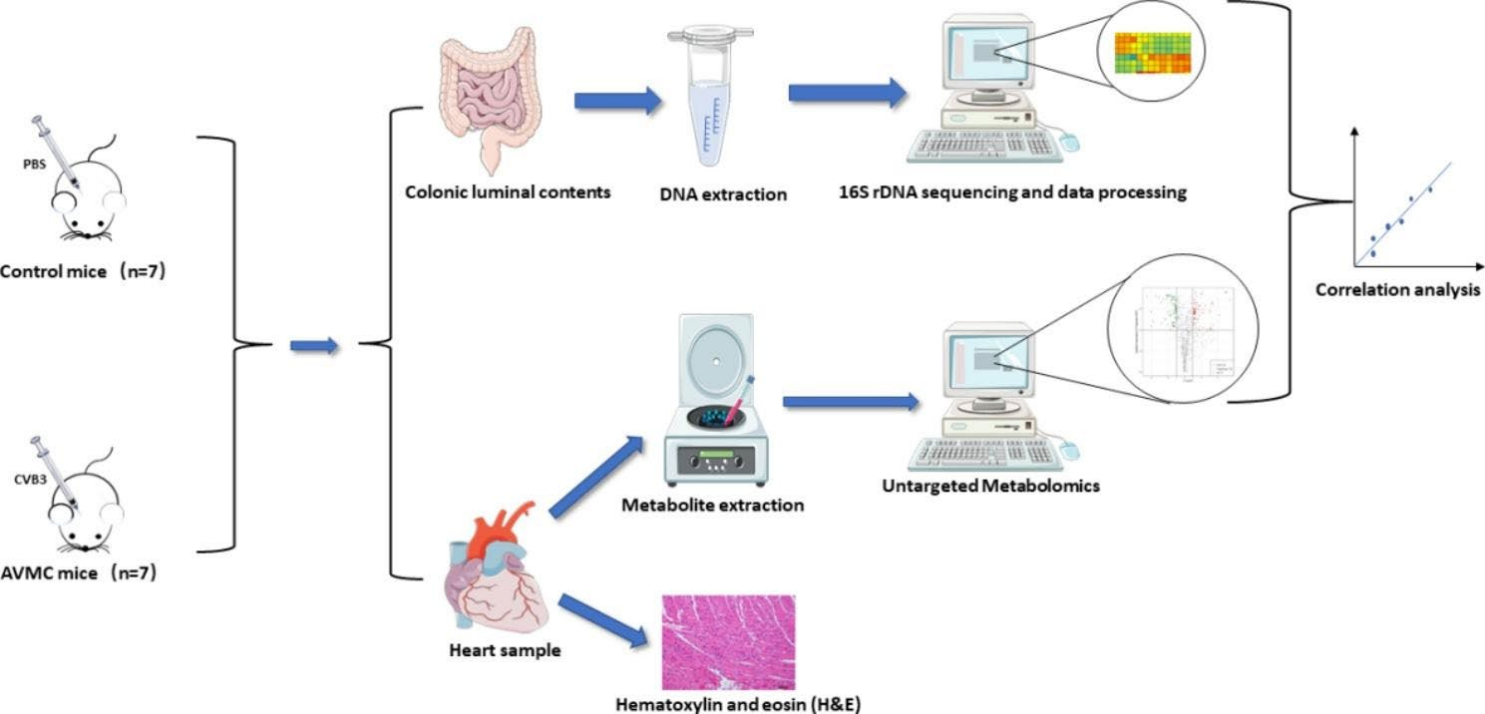
Schematic diagram of the experimental protocol involved in the collection of heart and colonic luminal contents samples from the mouse

Herein, we presented our study to firstly, systematically and comprehensively explore the gut microbiome and cardiac metabolome of mouse AVMC. 16 S ribosomal DNA (16 S rDNA) gene sequencing technology was used to identify changes of gut microbiome compositions. Moreover, untargeted ultra-performance liquid chromatography-tandem mass spectrometry (UPLC-MS/MS) was applied to capture the alteration of cardiac metabolites. Then the correlation between gut microbes and metabolites was revealed by correlation analysis (Fig. 1). The combined results based on these omics may help to decipher the association of AVMC with gut microbiota and cardiac metabolites, and provide novel insights into pathogenesis for AVMC.

## Methods

### Experimental animals

Our study was performed in accordance with protocols approved by the First Affiliated Hospital of Guangxi Medical University Ethical review Committee, China. Inbred male BALB/c mice (4–5 weeks of age) were purchased from Beijing Weitong Lihua Experimental Animal Technology (Beijing, China)[Permit Number: SCXK(JING)2016-0006]. All animals were housed and maintained in specific pathogen-free barrier facilities at the Laboratory Animal Center of Guangxi Medical University and were given standard drinking water and fodder. CVB3 was provided by microbiology Laboratory of Basic Medical College in Guangxi Medical University (Nanning, China).

All mice were randomly divided into two groups: Control mice (n = 7) and AVMC mice (n = 7). The establishment of AVMC mouse model performed as previously described [26]. Briefly, AVMC mice were established by a single intraperitoneal (i.p.) injection with 200 µL of CVB3(Nancy strain, median tissue culture infective dose TCID50 = 10^8^) diluted in phosphate buffered saline (PBS). At the same time, 200 µL PBS was injected to Control mice(i.p.). The day of injection was defined as day 0. Because the susceptible mouse strain, Balb/c, develops acute myocarditis around 10 to 14 days after infection [2], thus mice were killed under deep anesthesia by sodium phenobarbital on day 14. Hearts were collected and processed for untargeted metabolomic and histological analyses. To obtain the colonic luminal contents for microbiota composition, the entire colon was removed under sterile condition, and the outer surface of the intestine was washed with sterile water. Then colonic luminal contents were collected with sterile ophthalmic tweezer and placed in a sterile centrifuge tube for DNA extraction and microbiome analysis [27, 28].

### Histology

Hearts were fixed in 10% buffered formalin. Myocardial inflammation was assessed by Hematoxylin and eosin (H&E) as previously described [29]. The histological assessments were performed by two independent pathologists, who evaluated the percentage of the size of the heart section.

### DNA extraction and high-throughput 16 S rDNA gene sequencing for microbiome analysis

The genomic DNA of colonic luminal contents was extracted using the FastDNA® Spin Kit for Soil (MP Biomedicals, USCAT NO.116,560–200, Omega Bio-tek, Norcross, GA, U.S.) according to the manufacturer’s guidelines. After verified by 1% agarose gel electrophoresis, 16 S ribosomal DNA (rDNA)-based amplification was performed using the 515 F/806R primer set (5′ -GTGYCAGCMGCCGCGGTAA-3′ /5′ -GGACTACNVGGGTWTCTAAT-3′) with a PCR thermocycler (ABI, CA, USA), directionally targeting the V3 and V4 hypervariable regions of the 16 S rDNA gene. Then the PCR amplification of the 16 S rDNA genes were taken in triplicate and performed as follows: 95℃ for 3 min; 27 cycles of denaturing at 95℃ for 30 s, 55℃ for 30 s, and 72℃ for 45 s; a single extension at 72℃ for 10 min, and ending at 4℃. After extracted from 2% agarose gel and purified by the AxyPrep DNA Gel Extraction Kit (Axygen Biosciences, Union City, CA, USA), the PCR product was quantified by a Quantus™ Fluorometer (Promega, USA). Library was built using the TruSeq® DNA PCR-Free Sample Preparation Kit before sequencing. After Qubit and Q-PCR quantitation, the constructed library was qualified and sequenced by Novaseq6000.

### Processing of sequencing data

After truncating barcode and primer sequence, the reads of the samples were spliced with FLASH software(V1.2.11, http://ccb.jhu.edu/software/FLASH/) [30], then Raw Tags were obtained. Quality filtering on the raw tags were performed using the fastp (Version 0.20.0) software to obtain high-quality Clean Tags. These Clean Tags were compared with the reference database (Silva database https://www.arbsilva.de/) using Vsearch (Version 2.15.0) to detect the chimera sequences, and then the chimera sequences were removed to obtain the Effective Tags [31]. Then Effective Tags are denoised by using the QIIME2(Version QIIME2-202006), sequences with abundance less than 5 are filtered out, thus, the final ASVs (Amplicon Sequence Variants) were obtained. To obtain species information for each ASV, the ASVs were compared with the database(Silva 138.1), by using the classify-sklearn module in QIIME2 software.

By using QIIME2, alpha diversity including Chao1, Observed_features, faith_pd, Shannon_entropy and Simpson, were performed to analyze the richness and diversity in the sample. Weighted Unifrac and Unweighted Unifrac were used to analyze beta diversity and assess the diversity in samples for species complexity. R software (Version 3.5.3) was used to find out the significantly different species at different taxonomic level. Furthermore, Linear discriminant analysis effect size (LEfSe) analysis of species differ significantly between AVMC and Control groups, by using linear discriminant analysis (LDA) to estimate the effect of the abundance of each component (species) on the effect of the difference [32].

### Heart preparation and UPLC-MS/MS data acquisition for metabolomics analysis

After taken out from the − 80 °C refrigerator, 20 mg of heart sample were homogenized (30 HZ) for 20 s and the centrifuge (3000 rpm, 4 °C) for 30 s. After 400µL of 70% methanol water internal standard extractant were added, heart samples were shaked (1500 rpm) for 5 min and placed on ice for 15 min, and centrifuged (12,000 rpm, 4 °C) for 10 min. Then the supernatant (300 µL) was transferred and stayed at -20 °C for 30 min. After centrifuging (12,000 rpm, 4 °C) for 3 min, the 200 µL of supernatant was taken for analysis, by using high-performance liquid chromatography-electrospray tandem mass spectrometry (LC-ESI-MS/MS) system (UPLC, ExionLC AD; MS, QTRAP® 6500 + System, Sciex). Based on the metabolites eluted during this period, we annotated the metabolites through the self-built target standard database MWDB (metware database) by widely target UPLC-MS/MS platform of Metware Biotechnology Co., Ltd (Wuhan, China).

### UPLC-MS/MS-based metabolomics data analysis

By using statistics function prcomp within R (www.r-project.org), we performed Unsupervised PCA (principal component analysis). Heatmaps with dendrograms were used to display the HCA (hierarchical cluster analysis) results of samples and metabolites. VIP > = 1 and absolute Log2FC (fold change) > = 1 was used to identified significant changed metabolites between AVMC and Control groups. For VIP whose values were extracted from OPLS-DA result, it is generated by using R package MetaboAnalystR. And the OPLS-DA result contained score plots and permutation plots. Before OPLS-DA, the data was log transform (log2) and mean centering. The differential metabolites identified according to the screening criteria were annotated in KEGG database (http://www.kegg.jp/kegg/compound/), and classified according to the pathway types in KEGG (http://www.kegg.jp/kegg/pathway.html). Using a hypergeometric test’s p-value for a given list of metabolites, significantly enriched pathways were identified.

### Statistics analysis

All data were plotted as the average ± standard error of the mean. A p value of less than 0.05 was considered to be statistically significant. Wilcoxon rank-sum test was used to compare bacterial diversity and abundance between different groups. Heat maps were constructed based on the Wilcoxon rank-sum test at ASVs level. Correlation analysis of the gut microbiome and cardiac metabolites was performed by Spearman’s rank correlation. Correlation | r | > = 0.8 and P-value < 0.05 was statistically significant.

## Results

### Evaluation for the severity of AVMC

**Fig. 2:**
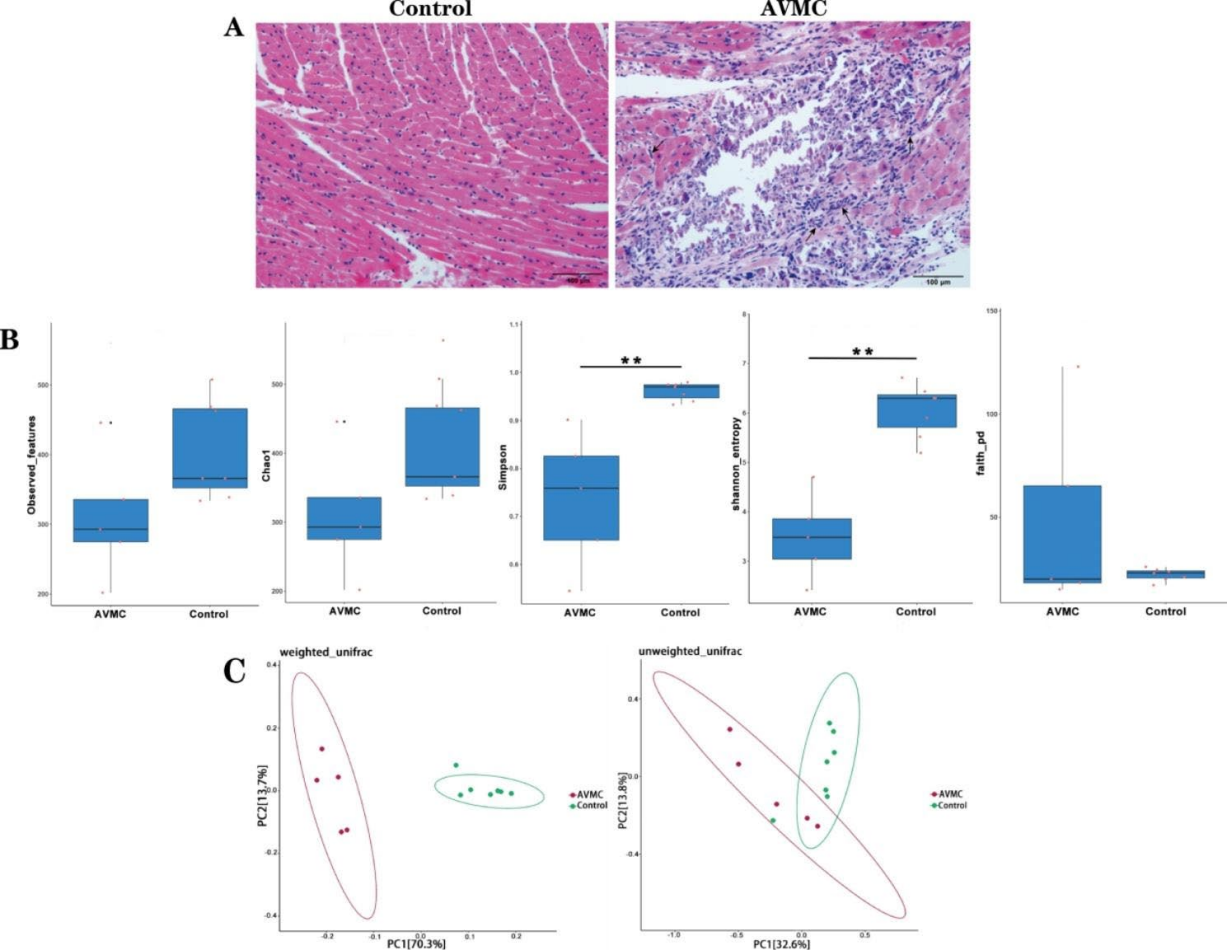
Gut microbial characteristics of AVMC and Control groups.(**A**):Representative of myocardial histopathologic images in AVMC and Control group (H&E, original magnification × 200). (**B**): Species diversity were estimated by the Observed_features, Chao1, Simpson, Shannon_entropy and faith_pd,indices; (**C**): PCoA plot based on the relative abundance of ASVs showed the bacterial structural clustering. Weighted UniFrac PCoA plots and Unweighted UniFrac PCoA plots were used. AVMC group (red dots); Control group (blue dots), where dots represent individual samples. AVMC, acute viral myocarditis. **p < 0.01, Control group (n = 7), AVMC group (n = 5)

Signs of AVMC were apparent in AVMC mice, including weight loss, weakness, anorexia, coat ruffling, irritability and lethargy. 2 of 7 mice died in the AVMC group, but no one died in the Control group. Compared with Control group, the cardiac pathological scores of AVMC were dramatically increased (2.6 ± 0.4, p < 0.05), because serious inflammatory cell infiltration was observed (Fig. 2A). The pathological scores were 0 in Control group.

### Decreased bacterial diversity in AVMC

In microbiome investigation, 2509 ASVs were obtained following taxonomic assignment (Supplementary Table S1). For Alpha diversity, levels of Chao1, Observed_features and faith_pd, were not significantly different, indicating the alteration of species richness indices between AVMC and Control groups were not significant. Compared with Control group, the AVMC group displayed significantly down-regulated levels of Shannon_entropy and Simpson (p < 0.05), indicating decreased species diversity in AVMC group (Fig. 2B). For Beta diversity, separation of the two groups was confirmed on the basis of the Weighted Unifrac PCoA (Fig. 2C).

### Alternations of colon microbial composition associated with AVMC

**Fig. 3:**
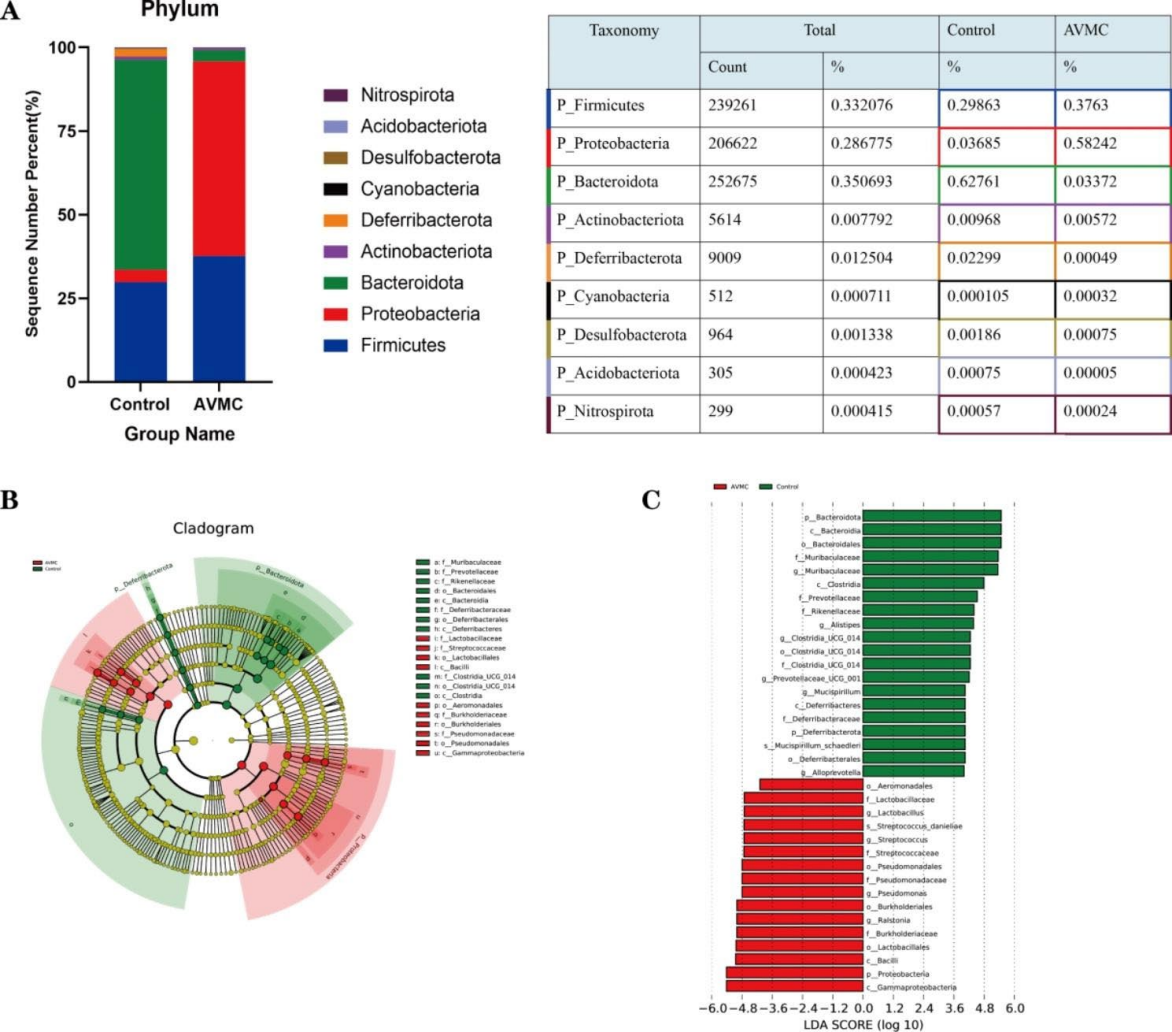
Gut microbiome structure analysis between AVMC and Control groups. (**A**): Component proportion of bacterial phylum in each group. (**B-C**) Linear discriminant analysis (LDA) integrated with effect size (LEfSe), (**B**): Cladogram indicating the phylogenetic distribution of microbiota correlated with the AVMC and Control groups, and (**C**): The differences in abundance between the AVMC and Control groups

The relative proportions of dominant taxa at the phylum level were assessed by microbial taxon assignment in colonic luminal contents. The most predominant phyla were Firmicutes, Bacteroidetes, Proteobacteria, Deferribacterota, and Actinobacteria. Compared with Control group, significant lower proportion of Bacteroidetes (62.76% vs. 3.37%, p < 0.01) and Deferribacterota (2.3% vs. 0.05%, p < 0.05) were observed in AVMC group. While higher proportion of Proteobacteria (3.69% versus 58.24%, p < 0.01) were observed in AVMC group. There was no significant change concerning other phyla groups including Firmicutes (29.86% versus 37.63%) and Actinobacteria (0.97% versus 0.57%) between the two groups (Fig. 3A). Compared with Control group, profoundly increased Firmicutes/Bacteroidetes ratio (F/B) was detected in AVMC group(0.51% vs. 14.63% p < 0.01). At genus level, higher proportion of Raistonia was confirmed in AVMC group (Figure S1).

**Fig. 4:**
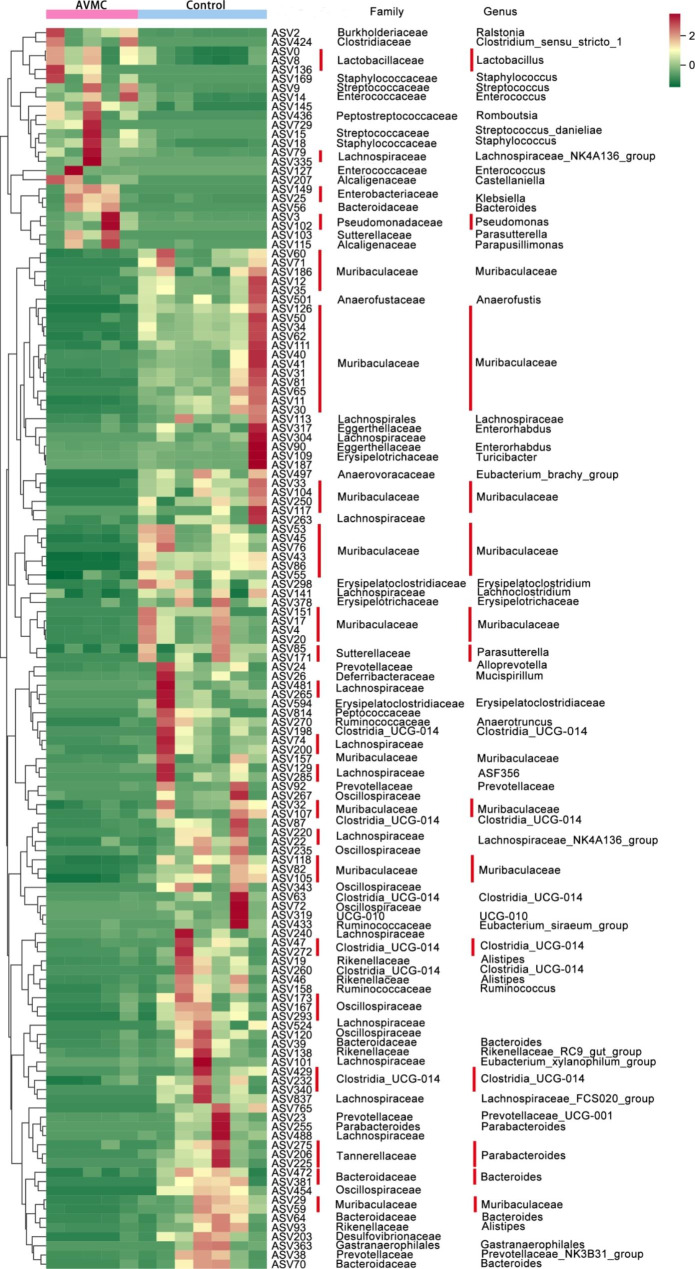
Heat map of the ASVs that differentiate the AVMC and Control groups. ASVs are shown from lower abundance (in green) to higher abundance (in red) for the z-transformed data. All 136 ASVs were assigned to families and genera

LEfSe was further performed to identify the specific bacteria associated with AVMC (Fig. 3B). Compared with the Control group, the relative abundance of 16 taxa in the AVMC group increased, while 20 taxa decreased (Fig. 3C). Among them, 10 opportunistic pathogens significantly overrepresented (all LDA scores (log10) > 4.8) in the AVMC group, including c__Gammaproteobacteria, p__Proteobacteria, c__Bacilli, o__Lactobacillales, f__Burkholderiaceae, g__Ralstonia, o__Burkholderiales, o__Pseudomonadales, f__Pseudomonadaceae, g__Pseudomonas. Whereas the Control mice primarily showed higher enrichment of Bacteroidales including p__Bacteroidota, c__Bacteroidia, o__Bacteroidales, f__Muribaculaceae, g__Muribaculaceae, c__Clostridia (LDA scores (log10) > 4.8). The relative abundances of these 136 ASVs were further analyzed by clustering analysis represented by a heat map (Fig. 4). The AVMC group was enriched with 24 ASVs, but the Control group was enriched with 112 ASVs.

### Global overview of cardiac metabolism in the AVMC and Control groups

**Fig. 5:**
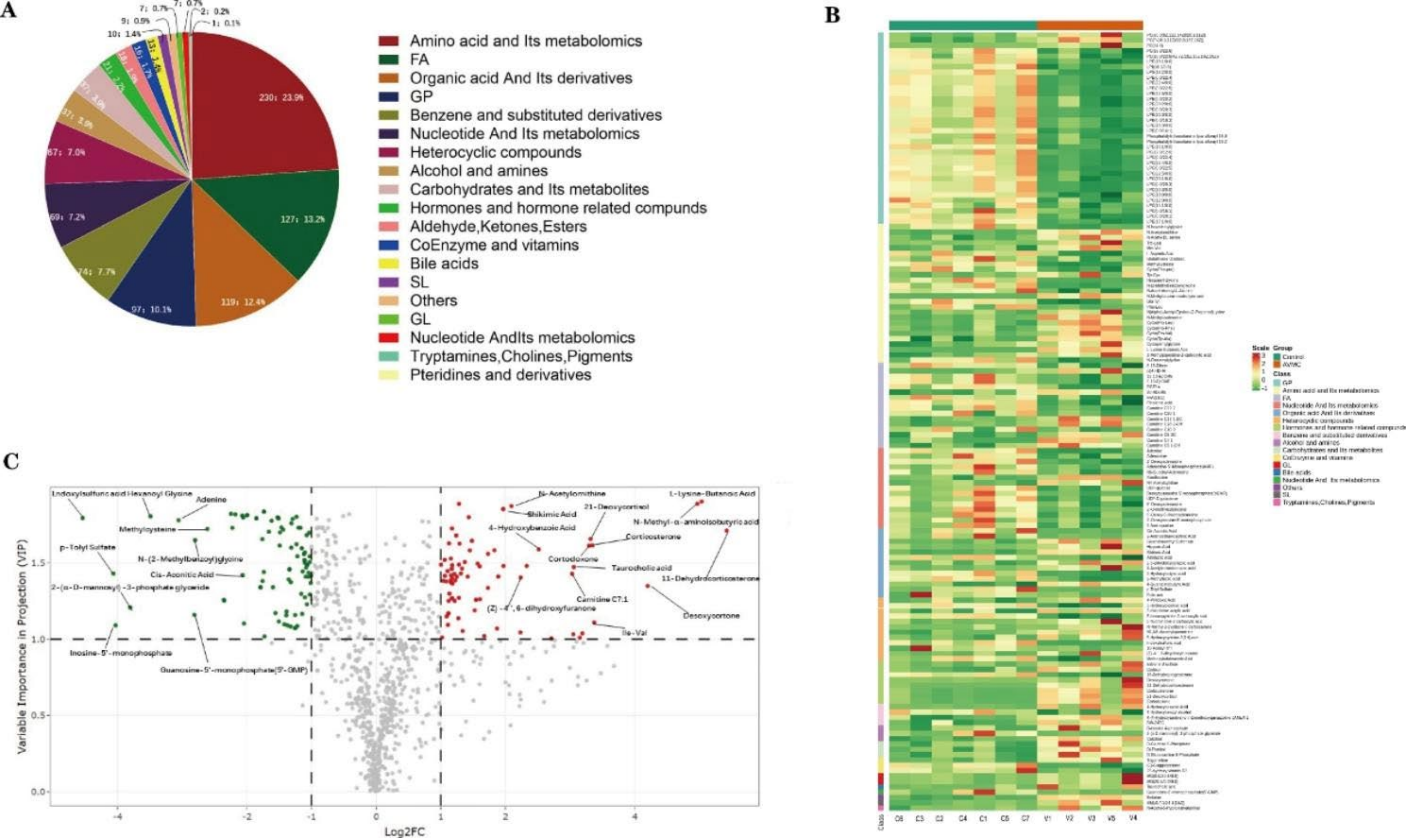
Cardiac metabolism characteristics of AVMC and Control groups. (**A**): The pie diagrams contain the percentages of identified metabolites classes in two groups. (**B**): Important discriminatory metabolites between the AVMC and Control groups. (**C**): Volcano plot of the differentially accumulated [log2 (fold-change) on X-axis] and significantly changed [VIP on Y-axis] metabolites

We subsequently performed metabolome analysis of heart samples using a UPLC-MS/MS-based nontargeted metabolomics. We identified 961 metabolites in the two groups, which were mapped onto 87 different KEGG metabolic pathways such as Carbohydrates metabolism, organic acid metabolism, and Biosynthesis of cofactors (Supplementary Table S2). Deferentially accumulated and significantly changed metabolites were classified into 19 compound groups, with the first 4 groups accounting for 59% of the total metabolites (Fig. 5A). Amino acids and metabolomics, which were the most abundant accounting for 24% of the total compounds, contained 230 metabolites. The fatty acyl class, Organic acids and the derivatives, Glycerophospholipids accounted for 13%, 12%, and 10%, respectively. Among the quantified 961 metabolites in the two groups, 146 metabolites were significant difference between AVMC and Control groups. Compared with Control group, 62 metabolites were increased while 84 were decreased in AVMC group(Supplementary Table S3).

### Clustering, correlation, and multivariate analysis reveal discriminatory metabolites between the AVMC and Control groups

We further performed HCA analysis on the metabolite abundances in the AVMC and Control groups, and the results displayed 17 large clusters (Fig. 5B). Concerning the metabolite clusters, significant difference was found between AVMC and Control groups, including organic acid and its derivatives, FA, Heterocyclic compounds, Hormones and hormone related compounds, Carbohydrates and its metabolites, Bile acids, CoEnzyme and vitamins, GL, Nucleotide and its metabolomics, Cholines and Pigments. Specifically, higher abundances of nucleotide and its metabolomics were observed in the Control group, such as Guanosine-5’-monophosphate (5’-GMP). Hormones and hormone related compounds such as Desoxycortone, 21-Deoxycortisol, were also overrepresented in the AVMC group (Fig. 5C). Meanwhile, a significant correlation was identified between these metabolites (Figure S2).

**Fig. 6:**
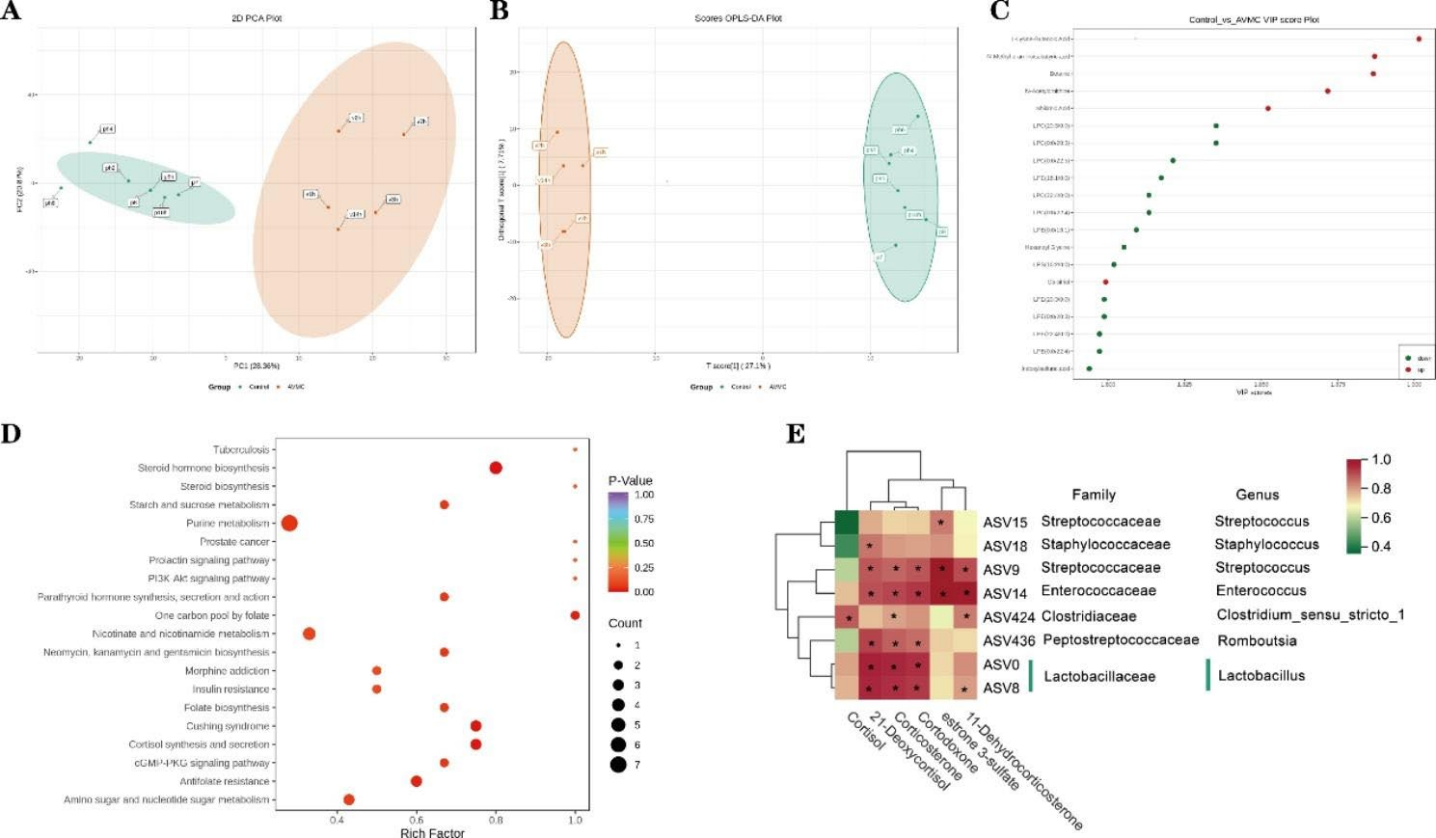
Metabolic profile as well as multivariate analysis and metabolic pathway of metabolites in AVMC and Control groups. (**A**): PCA analysis displaying the grouped discrimination of the AVMC and Control groups. (**B**): OPLS-Scoreplot: OPLS analysis displaying the group discrimination. (**C**): VIP scores of the important discriminatory metabolites obtained from the OPLS-DA models. (**D**): Differential metabolites enriched in 20 KEGG pathways. (**E**): Correlation analysis of the Steroid hormone biosynthesis related metabolites and gut microbiome. *p < 0.05, denoted statistical significance between bacterial taxa and metabolites

Based on the first two principal components, PC1 (28.36%) and PC2 (20.87%), metabolic differences between the AVMC and Control groups were confirmed by PCA analysis (Fig. 6A). Meanwhile, OPLS-DA analysis found several specific metabolites significantly enriched in AVMC group, such as L-Lysine-Butanoic Acid, Betaine, Shikimic Acid (Fig. 6B and C). On the other hand, higher abundances of metabolites in Control group were able to differentiate AVMC from Control groups, such as LPC (i.e., 20:3/0:0), LPE (i.e., 16:1/0:0), LPS (i.e., 16:2/0:0), and Hexanoyl Glycine (Fig. 6C). Pathway analysis was further used for these differential metabolites. Among 20 pathways, the Steroid hormone biosynthesis, Cortisol synthesis and secretion related metabolic pathway were most significantly enriched(Fig. 6D). Taken together, AVMC mice presented a specific cardiac metabolome in our study.

### Association of the dysregulation metabolites with gut microbial dysbiosis in AVMC

Steroid hormone biosynthesis related metabolic pathway was the most significantly enriched pathway. Moreover, its differential metabolites showed significant positive correlation with gut microbial 21-Deoxycortisol, Corticosterone, Cortodoxone and 11-Dehydrocorticosterone, were significantly positively correlated with Streptococcus, Enterococcus, Romboutsia, Lactobacillus (Fig. 6E). Notably, estrone 3-sulfate was significantly positively correlated with Streptococcus and Enterococcus. As the highest VIP score metabolite, L-Lysine-Butanoic Acid showed a negatic correlation with g_Ruminococcus_gnavu and g_Eubacterium_xylanophilum (Figure S3).

## Discussion

Enteroviruses CVB3, the dominant cause factor for AVMC, is taken into esophageal and gastrointestinal tracts via fecal-oral route [2]. Confirmed by clinical practice, myocarditis is considered as a rare extraintestinal complication of inflammatory bowel disease that can appear as an initial manifestation of Crohn’s disease [33]. Thus our current work aims to elucidate whether a causal link exists between CVB3-induced AVMC and gut. To our knowledge, our study is the first attempt to investigate the changes of gut microbiota and metabolome in an animal model of CVB3-induced AVMC.

Our results demonstrated profound changes of the gut microbiome, marked by altered composition and reduced diversity of the bacterial population in AVMC mice. The Control group was enriched with 112 ASVs, while the AVMC group was only enriched with 24 ASVs, and these differentially abundant microbiota were sufficient to differentiate the microbiota of normal and AVMC mouse. F/B ratio was considered as common hallmarks of heart disease. Higher F/B ratio of the AVMC group, suggests disordered physiological processes in AVMC. Dramatic decreased abundance of Bacteroidetes was observed in AVMC group. Cristina Gil-Cruz et al. reported that heart-specific CD4^+^ T cells in myocarditis can cross-react with the microbial components anti-Bacteroides IgG in the intestine [22], thus highlighting the possibility of transforming AVMC into a targetable disease by manipulation of the microbiome such as Bacteroidetes. Firmicutes, Bacteroidetes, Proteobacteria and Actinobacteria were the most dominant bacterial phyla in our AVMC mice. Interesting, we observed diverse structures of microbiota between the AVMC and EAM [23]. Those differences are mainly attributed to the different animal models of myocarditis, since EAM was induced by the cardiac myosin heavy chain, while AVMC was induced by enteroviruses CVB3 which is cardiotropic.

Similar to the result of EAM [23], LEfSe analysis confirmed higher proportion of f-Lactobacillaceae in AVMC mouse. F-Lactobacillaceae might play an important protective role in AVMC owing to its ability to improve ischaemia tolerance [34], raise left ventricular function [19], and help blood pressure regulation [35]. As one of the most abundant genera in the AVMC group, Ralstonia positively correlated well with enriched shikimic acid, negatively with hexanoyl glycine and lysophosphatidylethanolamine (LPE). Ralstonia species have been identified to utilize fatty acids for polyhydroxyalkanoates production [36],be associated with the pathogenesis of infective endocarditis [37]. Thus opportunistic pathogens Ralstonia may aggravate AVMC through metabolisms of Amino acid, Organic acid and GP. In addition, AVMC group was rich of Pseudomonas, which is considered as an enterotoxin-producing bacterium, and participating with kinds of intestinal and extra-intestinal infections [38]. Furthermore, Pseudomonas has shown a negative correlation with LPC, LPE and LPS [5], which is the major component of the outer membrane of Gram-negative bacteria and can trigger numerous downstream signaling processes [39]. Those data point to the potential effect of Pseudomonas on toxin production. Based on previous studies concerning gut dysbiosis and cardiovascular disease [24, 40], we hypothesized that microbiome alteration is an important cause of AVMC. Future experiments focusing on germ-free fecal transplantation would help to dissect cause from effect.

Dysbiosis of the gut microbiota in AVMC mouse was accompanied with the fluctuation of cardiac metabolome. The discriminating metabolites were mainly involved in GP, amino acid metabolism, FA, nucleotide metabolism and hormones and hormone related compounds. Compared with females, the incidence and seriousness of myocarditis is higher in males, which implied that sex differences was critical in AVMC [3, 2, 1]. In our study, metabolites about androgens, such as β-hydroxytestosterone, did not show significant change between AVMC and Control group. Contrary, estrone 3-sulfate significantly increased in AVMC mouse (FC = 7.59). Accumulating evidence confirmed Estrogen’s capacity to decrease cell-mediated immune responses and activate B cells, resulting in increased antibody responses to infection and autoantigens, while androgens have the opposite effect [41]. Therefore, high level of Estrogen may play an important protective role in AVMC. Importantly, estrone 3-sulfate showed a significantly positive correlation with Streptococcus and Enterococcus, indicating that supplement of these microbiota in gut might be a promising therapy for AVMC.

In the alteration of nucleotide and its metabolomics, the dramatic change in purine metabolism was confirmed by 7 increased metabolites, such as adenine and adenosine. Furthermore, steroid hormone biosynthesis pathway is significantly enriched in AVMC, with the highest enrichment density, including higher levels of 21-Deoxycortisol, Corticosterone, Cortodoxone and 11-Dehydrocorticosterone. Positive correlations were observed between these metabolomics and gut microbiota, including Streptococcus, Enterococcus, Romboutsia, and Lactobacillus. Clinical trials have shown the improvement of left ventricular ejection fraction after immunosuppression with azathioprine and prednisone [1, 42]. Thus purine and steroid hormone biosynthesis metabolism and their relative microbiota might be promising path way for immunosuppressive therapy to AVMC.

Of the total 146 deferentially expressed metabolites in AVMC mouse, 36 were involved in glycerophospholipids (GP) metabolism, mainly increased levels of LPC, LPS and LPE. These lysophospholipids (LPLs) were not only structural components of cellular membranes, but also biologically active molecules influencing a broad variety of processes such as immunity [43]. As a pro-inflammatory LPC, it is involved in modulation of T cell functions and immunity, triggering IL-1β processing and release [44], enhancing the expression of IFN-γ [45]. Therefore, the increased GP metabolism reprogramming may aggravate the development of AVMC.

Different from our previous data which obtained from sera of AVMC mouse and performed by NMR-based metabonomic analyses [29], our current data recovered that amino acid and its 15 metabolomics were all increased in AVMC, including betaine with high VIP score. As an intracellular osmolyte, betaine can prevent premature apoptosis [46, 47]. Higher baseline levels of betaine are associated with an increased risk of atrial fibrillation, heart failure and ischemic stroke [47, 48]. Furthermore, a positive correlation was found between betaine and g__Ruminococcus__gnavus_group, consistent with previous report that Ruminococcus increased the level of betaine [49]. Increased of taurocholic acid was seen in AVMC group. As an important bile acid which can decrease the expression of pro-inflammatory cytokines and chemokines [50], taurocholic acid may promote the healing of AVMC. As the main source of energy for cardiac mechanical work, fatty acid (FA) metabolomics such as FFA (16:1) were decreased in AVMC group, consistent with previous findings in bacterial myocarditis [51]. While Phosphate sugars metabolomics such as D-Glucose 6-Phosphate increased, indicating improvement of cardiomyocytes energy demand in AVMC.

## Conclusion

In summary, our findings provide evidence of microbial dysbiosis and metabolites dysregulation in AVMC, suggest gut microbiome may participate in the development of AVMC, the mechanism may be related to metabolism reprogramming involving several pathways such as hormones and hormone related compounds. Our findings provide a new perspective for understanding the pathogenesis and directions of therapies for AVMC.

## Electronic supplementary material

Below is the link to the electronic supplementary material.

Additional file 1.

Additional file 2.

## Data Availability

The raw data of 16s rDNA presented in this study were deposited in NCBI Sequence Read Archive(SRA) under primary accession PRJNA845097 and the accession numbers are SRR19547015、SRR19547016、SRR19547017、SRR19547018、SRR19547019、SRR19547022、SRR19547023 for control, SRR19547012、SRR19547013、SRR19547014、SRR19547020、SRR19547021 for AVMC.

## Abbreviations

AVMC: Acute viral myocarditis
DCM: Dilated cardiomyopathy
CVB3: Coxsackievirus group B type 3
EAM: Experimental autoimmune myocarditis
16S rDNA: 16S ribosomal DNA
UPLC-MS/MS: Untargeted ultra-performance liquid chromatography-tandem mass spectrometry
PBS: Phosphate buffered saline
H&E: Hematoxylin and eosin
ASVs: Amplicon Sequence Variants
LEfSe: Linear discriminant analysis effect size
LDA: Linear discriminant analysis
PCA: Principal component analysis
HCA: Hierarchical cluster analysis
F/B: Firmicutes/Bacteroidetes ratio
GP: Glycerophospholipids
LPC: Lysophosphatidylcholine
LPE: Lysophosphatidylethanolamine
LPS: Lipopolysaccharide

## References

[1] Olejniczak M, Schwartz M, Webber E, Shaffer A, Perry T (2020). Viral Myocarditis-Incidence, diagnosis and management. J Cardiothorac Vasc Anesth.

[2] Lasrado N, Reddy J (2020). An overview of the immune mechanisms of viral myocarditis. Rev Med Virol.

[3] Narovlyanskaya O, Winokur EV, Myocarditis (2020). Dimens Crit Care Nurs.

[4] Qian Q, Xiong S, Xu W (2012). Manipulating intestinal immunity and microflora an alternative solution to viral myocarditis. Future Microbiol.

[5] Giuliani C. The Flavonoid Quercetin Induces AP-1 Activation in FRTL-5 Thyroid Cells. Antioxid (Basel) 2019, 8. 10.3390/antiox8050112 PMC6562732 31035637

[6] Slavich GM, Social Safety Theory (2020). A biologically based evolutionary perspective on life stress, Health, and Behavior. Ann Rev Clin Psychol.

[7] Collins SM (2014). A role for the gut microbiota in IBS. Nat Rev Gastroenterol Hepatol.

[8] Zhao Y, Wang Z (2020). Gut microbiome and cardiovascular disease. Curr Opin Cardiol.

[9] Adak A, Khan M (2019). An insight into gut microbiota and its functionalities. Cell Mol Life Sci.

[10] Eloe-Fadrosh EA, Rasko DA (2013). The human microbiome: from symbiosis to pathogenesis. Annu Rev Med.

[11] Zhang Y, Wang Y, Ke B (2021). J. TMAO: how gut microbiota contributes to heart failure. Transl Res.

[12] Swanepoel I, Roberts A, Brauns C, Chaliha DR, Papa V, Palmer RD (2022). Trimethylamine N-oxide (TMAO): a new attractive target to decrease cardiovascular risk. Postgrad Med J.

[13] Bartolomaeus H, Balogh A, Yakoub M, Homann S, Markó L, Höges S (2019). Short-chain fatty acid Propionate protects from Hypertensive Cardiovascular damage. Circulation.

[14] Feng Y, Cao G, Chen D, Vaziri N, Chen L, Zhang J (2019). Microbiome-metabolomics reveals gut microbiota associated with glycine-conjugated metabolites and polyamine metabolism in chronic kidney disease. Cell Mol Life Sci.

[15] Yang Y, Misra BB, Liang L, Bi D, Weng W, Wu W (2019). Integrated microbiome and metabolome analysis reveals a novel interplay between commensal bacteria and metabolites in colorectal cancer. Theranostics.

[16] Park Y, Lee S, Kang M, Kim B, Lee M, Jung S (2020). Imbalance of gut Streptococcus, Clostridium, and Akkermansia determines the natural course of atopic dermatitis in infant. Allergy Asthma Immunol Res.

[17] Barrington WT, Lusis AJ, Atherosclerosis (2017). Association between the gut microbiome and atherosclerosis. Nat Rev Cardiol.

[18] Verhaar BJH, Prodan A, Nieuwdorp M, Muller M. Gut Microbiota in Hypertension and Atherosclerosis: A Review. Nutrients 2020, 12. 10.3390/nu12102982 PMC7601560 33003455

[19] Tang WHW, Li DY, Hazen SL (2019). Dietary metabolism, the gut microbiome, and heart failure. Nat Rev Cardiol.

[20] Tang WH, Kitai T, Hazen SL (2017). Gut microbiota in Cardiovascular Health and Disease. Circ Res.

[21] Wang Z, Zhao Y (2018). Gut microbiota derived metabolites in cardiovascular health and disease. Protein Cell.

[22] Gil-Cruz C, Perez-Shibayama C, De Martin A, Ronchi F, van der Borght K, Niederer R (2019). Microbiota-derived peptide mimics drive lethal inflammatory cardiomyopathy. Science.

[23] Hu XF, Zhang WY, Wen Q, Chen WJ, Wang ZM, Chen J (2019). Fecal microbiota transplantation alleviates myocardial damage in myocarditis by restoring the microbiota composition. Pharmacol Res.

[24] Pollack A, Kontorovich AR, Fuster V, Dec GW (2015). Viral myocarditis–diagnosis, treatment options, and current controversies. Nat Rev Cardiol.

[25] Jr CL, Myocarditis. N Engl J Med. 2009;360:1526–38. 10.1056/NEJMra0800028 PMC5814110 19357408

[26] Kong Q, Wu W, Yang F, Liu Y, Xue Y, Gao M et al. Increased Expressions of IL-22 and Th22 cells in the coxsackievirus B3-Induced mice acute viral myocarditis. Virol 2012, 9. 10.1186/1743-422X-9-232 PMC3544697 23050732

[27] Kok DEG, Rusli F, van der Lugt B, Lute C, Laghi L, Salvioli S (2018). Lifelong calorie restriction affects indicators of colonic health in aging C57Bl/6J mice. J Nutr Biochem.

[28] van der Lugt B, Lute RF, Lamprakis C, Salazar A, Boekschoten E, Hooiveld MV, Müller GJ, Vervoort M, Kersten J, Belzer S, Kok C, Steegenga DEG (2018). Integrative analysis of gut microbiota composition, host colonic gene expression and intraluminal metabolites in aging C57BL/6J mice. Aging.

[29] Kong Q, Gu J, Lu R, Huang C, Hu X, Wu W et al. NMR-Based Metabolomic Analysis of Sera in Mouse Models of CVB3-Induced Viral Myocarditis and Dilated Cardiomyopathy. Biomolecules 2022, 12. 10.3390/biom12010112 PMC8773787 35053260

[30] Magoc T, Salzberg SL (2011). FLASH: fast length adjustment of short reads to improve genome assemblies. Bioinformatics.

[31] Haas BJ, Gevers D, Earl AM, Feldgarden M, Ward DV, Giannoukos G (2011). Chimeric 16S rRNA sequence formation and detection in Sanger and 454-pyrosequenced PCR amplicons. Genome Res.

[32] Segata N, Izard J, Waldron L, Gevers D, Miropolsky L, Garrett WS et al. Metagenomic biomarker discovery and explanation. Genome Biol 2011, 12. 10.1186/gb-2011-12-6-r60 PMC3218848 21702898

[33] Leuschner F, Katus HA, Kaya Z (2009). Autoimmune myocarditis: past, present and future. J Autoimmun.

[34] Lam V, Su J, Koprowski S, Hsu A, Tweddell JS, Rafiee P (2012). Intestinal microbiota determine severity of myocardial infarction in rats. FASEB J.

[35] Khalesi S, Sun J, Buys N, Jayasinghe R (2014). Effect of probiotics on blood pressure: a systematic review and meta-analysis of randomized, controlled trials. Hypertension.

[36] Bhatia SK, Gurav R, Choi TR, Jung HR, Yang SY, Song HS (2019). Poly(3-hydroxybutyrate-co-3-hydroxyhexanoate) production from engineered Ralstonia eutropha using synthetic and anaerobically digested food waste derived volatile fatty acids. Int J Biol Macromol.

[37] Orme J, Rivera-Bonilla T, Loli A, Blattman NN. Native Valve Endocarditis due to Ralstonia pickettii: A Case Report and Literature Review. Case Rep Infect Dis 2015, 2015, 324675. 10.1155/2015/324675 PMC4306225 25648998

[38] Grover S, Batish V, Srinivasan R (1990). Production and properties of crude enterotoxin of Pseudomonas aeruginosa. Int J Food Microbiol.

[39] Medzhitov R (2007). Recognition of microorganisms and activation of the immune response. Nature.

[40] Cook KL (2021). Gut dysbiosis and hypertension: is it cause or effect?. J Hypertens.

[41] Fairweather D, Cooper LT, Blauwet LA (2013). Sex and gender differences in myocarditis and dilated cardiomyopathy. Curr Probl Cardiol.

[42] Wojnicz R, Nowalany-Kozielska E, Wojciechowska C, Glanowska G, Wilczewski P, Niklewski T (2001). Randomized, placebo-controlled study for immunosuppressive treatment of inflammatory dilated cardiomyopathy: two-year follow-up results. Circulation.

[43] Grzelczyk A, Gendaszewska-Darmach E (2013). Novel bioactive glycerol-based lysophospholipids: new data -- new insight into their function. Biochimie.

[44] Stock C, Schilling T, Schwab A, Eder C (2006). Lysophosphatidylcholine stimulates IL-1beta release from microglia via a P2 × 7 receptor-independent mechanism. J Immunol (Baltimore Md : 1950).

[45] Nishi E, Kume N, Ueno Y, Ochi H, Moriwaki H, Kita T (1998). Lysophosphatidylcholine enhances cytokine-induced interferon gamma expression in human T lymphocytes. Circ Res.

[46] Xie L, Zhao BX, Luo J, Li Y, Zhu F, Li GF (2020). A U-shaped association between serum betaine and incident risk of first ischemic stroke in hypertensive patients. Clin Nutr.

[47] Slow S, Lever M, Chambers S, George P (2009). Plasma dependent and independent accumulation of betaine in male and female rat tissues. Physiol Res.

[48] Papandreou C, Bullo M, Hernandez-Alonso P, Ruiz-Canela M, Li J, Guasch-Ferre M (2021). Choline Metabolism and Risk of Atrial Fibrillation and Heart failure in the PREDIMED Study. Clin Chem.

[49] Koistinen VM, Karkkainen O, Borewicz K, Zarei I, Jokkala J, Micard V (2019). Contribution of gut microbiota to metabolism of dietary glycine betaine in mice and in vitro colonic fermentation. Microbiome.

[50] Levi M (2016). Role of bile acid-regulated nuclear receptor FXR and G protein-coupled receptor TGR5 in regulation of Cardiorenal Syndrome (Cardiovascular Disease and chronic kidney disease). Hypertension.

[51] Yang C, Zhao K, Chen X, Jiang L, Li P, Huang P (2021). Pellino1 deficiency reprograms cardiomyocytes energy metabolism in lipopolysaccharide-induced myocardial dysfunction. Amino Acids.

