# Supplementary material for: Alterations of the gut microbiome and metabolic profile in CVB3-induced mice acute viral myocarditis
Source: BMC Microbiol. 2023 May 18;23:139. doi: 10.1186/s12866-023-02863-4 (PMC10193676; doi:10.1186/s12866-023-02863-4)
Supplement: Supplementary file 2 — Additional file 2. [file 12866_2023_2863_MOESM2_ESM.docx]

Figure S1


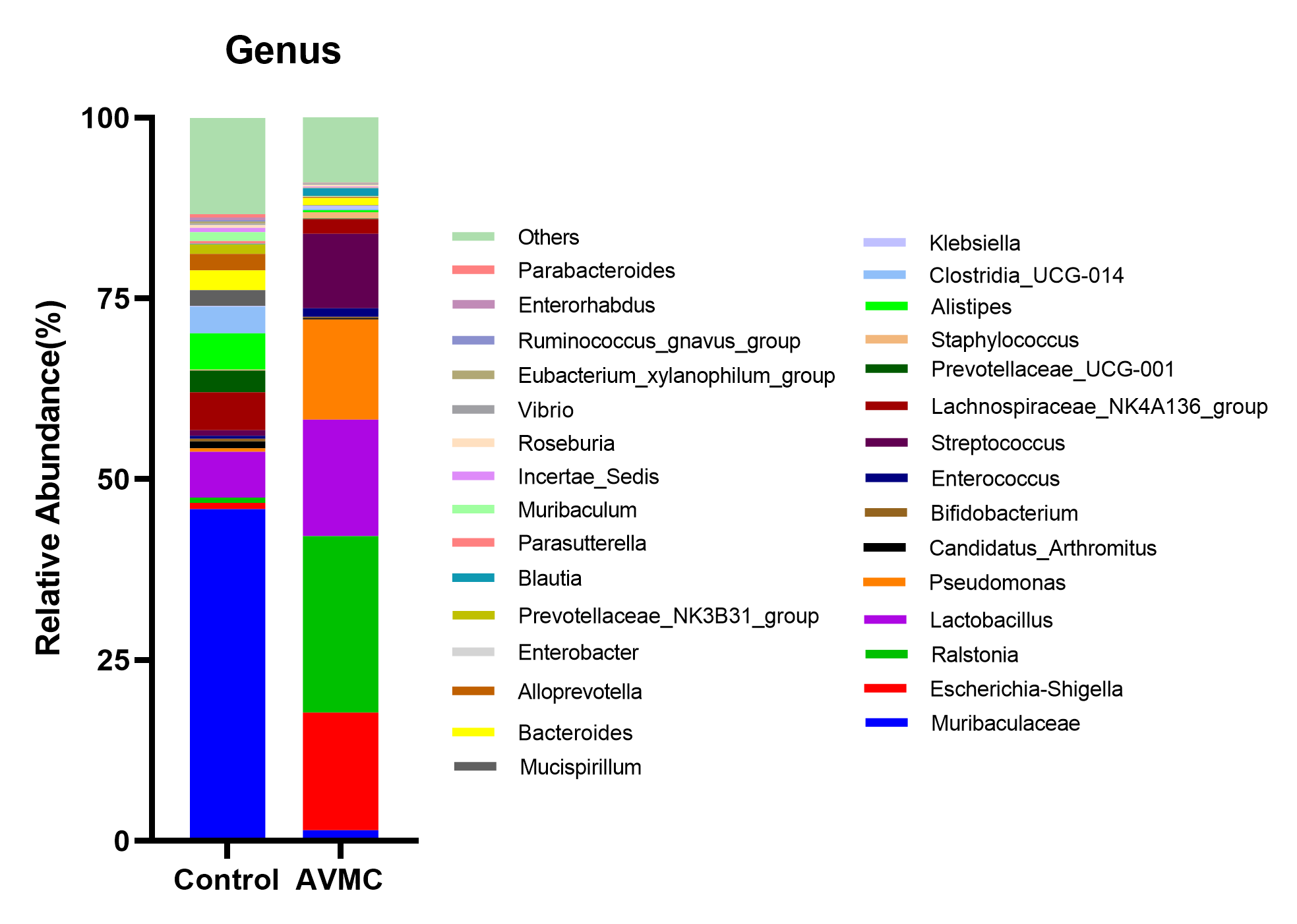


Figure S1：(**A**) Component proportion of bacterial at Genus levels.

Figure S2


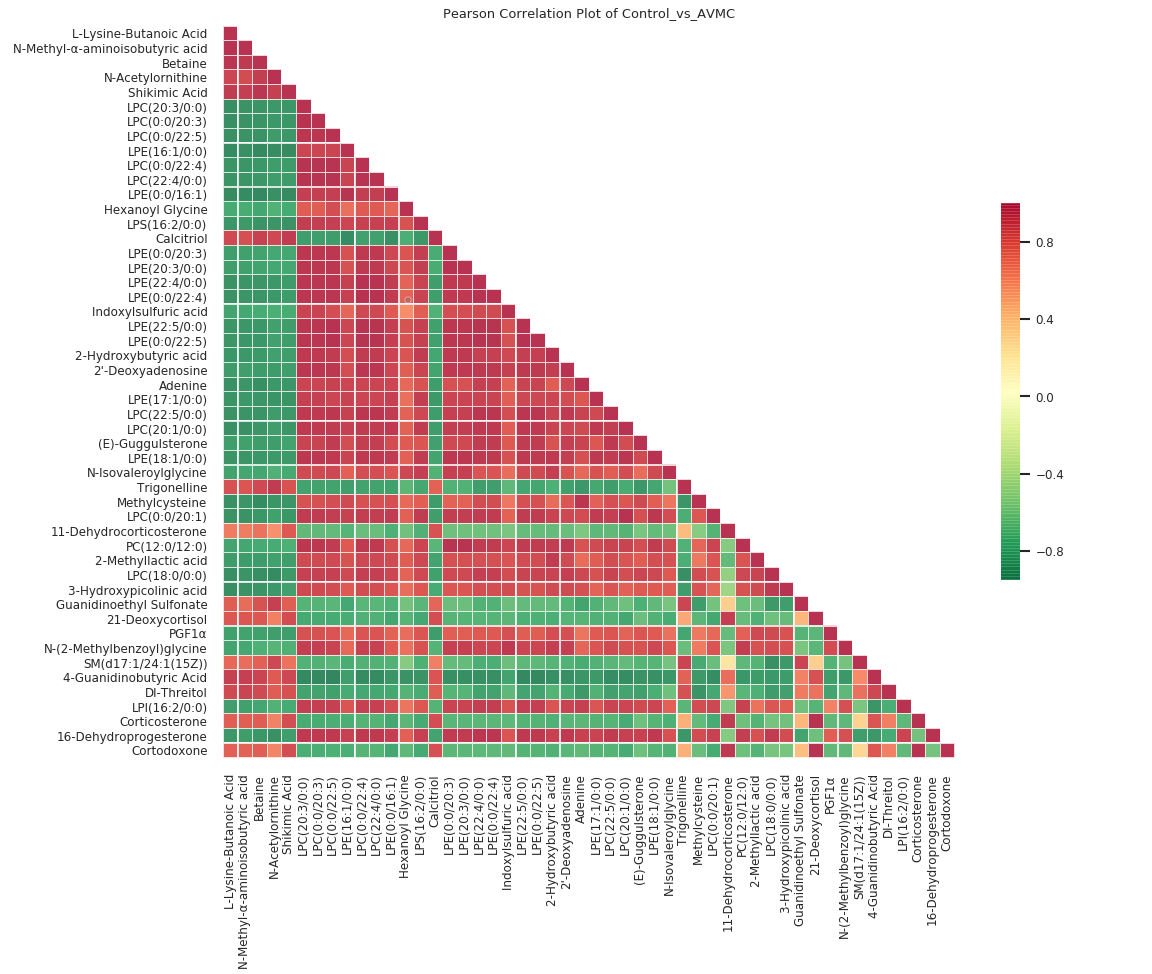
Figure S2: Top 50 VIP of Metabolite-metabolite (Pearson’s r) correlations. High (1, red) and low (0, green) correlations are shown.

Figure S3: Correlation analysis of gut microbiome and cardiac metabolites.


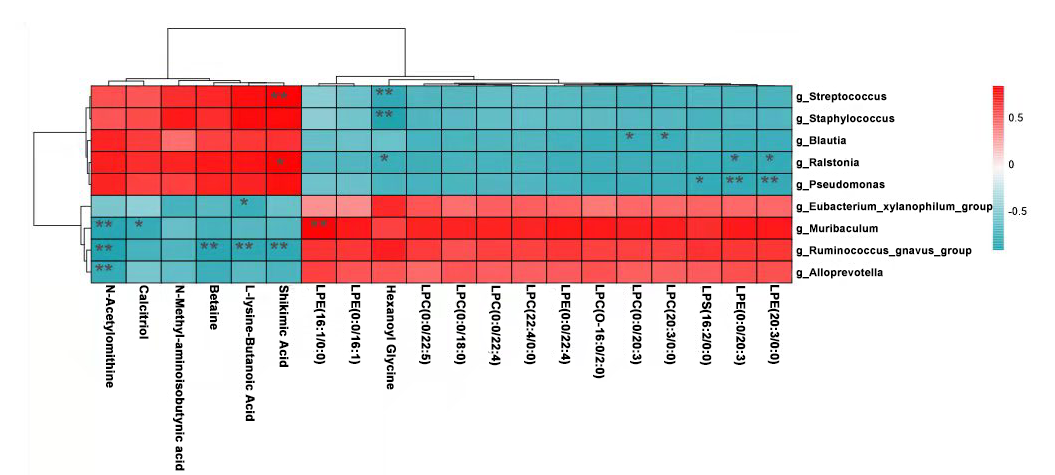


Figure S3: Spearman’s rank correlation between 9 most differential microbial genera and 20 differential metabolites with the top VIP. *p<0.05, **p<0.01 denoted statistical significance between bacterial taxa and metabolites.
